# Active immunization with human interleukin-15 induces neutralizing antibodies in non-human primates

**DOI:** 10.1186/s12865-016-0168-6

**Published:** 2016-09-26

**Authors:** Yunier Rodríguez-Álvarez, Yanelys Morera-Díaz, Haydee Gerónimo-Pérez, Jorge Castro-Velazco, Rafael Martínez-Castillo, Pedro Puente-Pérez, Vladimir Besada-Pérez, Eugenio Hardy-Rando, Araceli Chico-Capote, Klaudia Martínez-Cordovez, Alicia Santos-Savio

**Affiliations:** 1 Pharmaceutical Division, Center for Genetic Engineering and Biotechnology, Avenue 31, PO Box 6162, Havana, 10 600 Cuba; 2 Quality Control Division, Center for Genetic Engineering and Biotechnology, Avenue 31, PO Box 6162, Havana, 10600 Cuba; 3 Animal Facility Department, Center for Genetic Engineering and Biotechnology, Avenue 31, PO Box 6162, Havana, 10600 Cuba; 4 Chemistry and Physics Division, Center for Genetic Engineering and Biotechnology, Avenue 31, PO Box 6162, Havana, 10600 Cuba; 5 Biotechnology Laboratory, Study Center for Research and Biological Evaluations, Institute of Pharmacy and Foods, Havana University, Avenue 222, PO Box 13600, Havana, 10600 Cuba; 6 Rheumatology Department, Hermanos Ameijeiras Hospital, San Lazaro 701, PO Box 6122, Havana, 10600 Cuba

**Keywords:** IL-15, Cytokine, Neutralizing Abs, Immunization, Alum, Non-human primates, CTLL-2 cells

## Abstract

**Background:**

Interleukin-15 is an immunostimulatory cytokine overexpressed in several autoimmune and inflammatory diseases such as Rheumatoid Arthritis, psoriasis and ulcerative colitis; thus, inhibition of IL-15-induced signaling could be clinically beneficial in these disorders. Our approach to neutralize IL-15 consisted in active immunization with structurally modified human IL-15 (mhIL-15) with the aim to induce neutralizing antibodies against native IL-15. In the present study, we characterized the antibody response in *Macaca fascicularis*, non-human primates that were immunized with a vaccine candidate containing mhIL-15 in Aluminum hydroxide (Alum), Montanide and Incomplete Freund’s Adjuvant.

**Results:**

Immunization with mhIL-15 elicited a specific antibodies response that neutralized native IL-15-dependent biologic activity in a CTLL-2 cell proliferation assay. The highest neutralizing response was obtained in macaques immunized with mhIL-15 adjuvanted in Alum. This response, which was shown to be transient, also inhibited the activity of simian IL-15 and did not affect the human IL-2-induced proliferation of CTLL-2 cells. Also, in a pool of synovial fluid cells from two Rheumatoid Arthritis patients, the immune sera slightly inhibited TNF-α secretion. Finally, it was observed that this vaccine candidate neither affect animal behavior, clinical status, blood biochemistry nor the percentage of IL-15-dependent cell populations, specifically CD56^+^ NK and CD8^+^ T cells.

**Conclusion:**

Our results indicate that vaccination with mhIL-15 induced neutralizing antibodies to native IL-15 in non-human primates. Based on this fact, we propose that this vaccine candidate could be potentially beneficial for treatment of diseases where IL-15 overexpression is associated with their pathogenesis.

## Background

Cytokines are defined as short-range protein messengers with important functions in the regulation of the immune response and intercellular communications [1]. These proteins have been shown to be overexpressed in the context of several diseases, including allergies, autoimmune disorders, cancer and some infectious diseases [2–5]. A large number of therapeutic approaches aimed at inhibiting the activity of these molecules has been developed, and there are over than 20 cytokine-targeting pharmaceutical agents currently approved for clinical use [6].

Interleukin (IL)-15, one of the members of this protein family, is a pro-inflammatory cytokine that is overexpressed in several inflammatory disorders such as Rheumatoid Arthritis (RA), psoriasis, ulcerative colitis and sarcoidosis [7–11]. The participation of IL-15 in the pathogenesis of autoimmune diseases has been demonstrated by in vitro studies [12, 13], murine animal models [14] and clinical trials with an anti-human IL-15 antibody (Ab), AMG714 [15]. In particular, IL-15 is an important player in the inflammatory processes of RA, where it recruits circulating memory T cells in the synovial membrane and may up regulate other pro-inflammatory cytokines through a variety of mechanisms [16, 17]. Among these mechanisms are included the induction of tumour necrosis factor alpha (TNF-α) production through the activation of synovial T cells and macrophages via a cell contact-dependent mechanism [18], as well as the activation of Th17 lymphocytes driving up the biosynthesis of IL-17 [19].

One possible approach for inhibiting the activity of cytokines produced at pathogenic levels would be to actively immunize patients with the relevant cytokine coupled to a carrier protein or with the modified cytokine [20, 21]. This strategy, aimed at inducing high titers of neutralizing polyclonal auto-Abs against a pathogenic cytokine in order to antagonize its harmful effects without interfering with other physiologic processes [22, 23], would possibly exhibit less adverse events than passive immunization strategies, requiring much smaller number of doses, having a lower cost and not presenting the potential problem of anti-Ab response [24, 25]. Extensive overviews of anti-cytokines vaccination have been published which based almost on the results obtained in animal models, demonstrate that cytokine vaccination may be an effective solution to the control of several autoimmune diseases [26–31]. These data also suggest, based on the absence of severe side effects, that active anti-cytokine immunization represents a relatively safe solution [32]. Taking into account these promising results, new clinical trials have been developed using *kinoids*, which are approved by the Food and Drug Administration for clinical human use [33].

The aim of the current research was to induce a neutralizing Ab response against self-IL-15 as a potential therapeutic strategy for diseases involving the overexpression of this cytokine. Although an anti-human IL-15 Ab, AMG714, has already been tested in clinical trials [15], the cytokine itself has never been employed as a target for active vaccination. For our work, human IL-15 was expressed in *Escherichia coli* following the procedure described by Santos et al. [34]. The purified protein, denominated here as modified human IL-15 (mhIL-15), exhibits a scrambled disulfide bonds pattern and it has an additional Alanine residue at its N-terminus. In the present study, non-human primates (NHP) *Macaca fascicularis* were immunized with mhIL-15 adjuvanted in aluminum hydroxide (Alum), Montanide ISA-51 VG or Incomplete Freund’s Adjuvant (IFA). The immune response of the monkeys was analyzed by serum antigen-specific Ab titers and the neutralizing capacity of the resulting sera was determined in CTLL-2, an IL-15-dependent cell line. The effect of these sera on the biological activity of human IL-2 and simian IL-15 was also explored. Additionally, we examined the effect of sera from macaques immunized with Alum-adjuvanted mhIL-15 on IL-15-mediated TNF-α production by synovial fluid cells from patients with RA. Finally, the effect of immunization with mhIL-15 on IL-15-dependent cell populations was studied.

## Methods

### Animals. Handling and husbandry

Twelve adult macaques (*Macaca fascicularis*) of either sex were used, weighting from 2 to 5 kg. All animals were purchased from the National Center for Animal Breeding (CENPALAB, Havana, Cuba) and maintained in the animal facility of the Center for Genetic Engineering and Biotechnology (CIGB, Havana, Cuba). An environmental temperature of 22–29 °C and a light/dark cycle of 12:12 h were maintained throughout the study. The animals were housed individually in stainless steel cages (90 × 60 × 60 cm) and randomized into groups of 3 to receive mhIL-15 in each adjuvant. Three animals were used as control (placebo group).

The monkeys were adapted to laboratory conditions for at least 4 weeks. They were fed with fresh tomatoes, guavas, bananas, and commercial chow (certificated granulated formula CMQ 1600 ALYco; CENPALAB, Havana, Cuba, containing 25 % of proteins, 3.5 % of crude fat and 3.8 % of crude fiber) twice a day with 150–300 g per monkey according to their ages and body weights. Water was provided ad libitum. The animals were anesthetized with an intramuscular injection of 10 mg/kg ketamine hydrochloride (Liorad Laboratories, Havana, Cuba) before immunization. Vital signs, temperature, heart rate, blood pressure and body weight were registered along the whole scheme before each immunization.

### CTLL-2 cell line

CTLL-2 is a T cell-derived, IL-2 dependent cell line obtained from C57bl/6 mice. These cells were grown in RPMI medium 1640 (Thermo Fisher Scientific, USA) containing 2 mM L-glutamine (Thermo Fisher Scientific, USA), 50 μg/mL gentamicin (Sigma-Aldrich, USA), 10 % heat-inactivated fetal bovine serum (FBS, Capricorn Scientific, Germany) and 10 ng/ml recombinant human IL-2 (R&D, USA). Cells were incubated at 37 °C with 5 % CO_2_, 95 % humidity. CTLL-2 cells were harvested and used in log phase growth (Cell passage 5 after thawing; Cell viability: ≥95 %). Prior to use, the cells were washed 5 times with RPMI medium. The CTLL-2 cell bank was generated from cells directly obtained from ATCC (TIB-214).

### Purification of the recombinant mhIL-15

Expression of recombinant mhIL-15 in *E. coli* resulted in the formation of insoluble inclusion bodies. After extraction in buffer containing 8 M urea (Merck, USA) in phosphate-buffered saline (PBS, pH 7.4, Thermo Fisher Scientific, USA), mhIL-15 was purified using size-exclusion chromatography (SEC) followed by reverse phase (RP) - high performance liquid chromatography (HPLC). For SEC, we used a HiLoad 26/600 Superdex 200 preparative grade (60 cm × 26 mm, 34 μm, GE Healthcare, USA) column, which was operated at 4 mL/min. The mhIL-15-containing fraction, detected at 226 nm, was then loaded at 0.2 mL/min onto a C_4_ column (1 × 25 cm, 10 μm, Vydac, USA). The proteins were separated using a mobile phase containing 0.1 % trifluoroacetic acid (Sigma-Aldrich, USA) and HPLC grade acetonitrile (Sigma-Aldrich, USA), with 0–80 % acetonitrile gradient over 70 min at 2.5 mL/min. The separation was monitored at 226 nm [34].

### Enzyme digestion of purified mhIL-15

Twenty micrograms of RP-HPLC purified mhIL-15 was suspended in 20 μL of 50 mM NH_4_HCO_3_ (Sigma-Aldrich, USA) pH 8.0 and incubated for 4 h at 37 °C with trypsin (Promega, USA) 1:100 (w/w) enzyme: mhIL-15 ratio. Afterwards, endoproteinase Glu-C (Roche Biochemical Reagents, USA) was added in tandem, in the same ratio as trypsin, and incubated for 2 h at 37 °C. Peptides were desalted by ZipTips (Millipore, USA), eluted in 3 μL of 60 % acetonitrile (Sigma-Aldrich, USA) in 1 % formic acid (Caledon, Canada) and injected in a hybrid quadrupole-time-of-flight (QTOF-2) mass spectrometer (Micromass, UK).

### Mass spectrometry

Intact protein samples as well as peptide digests were analyzed by nano-electrospray ionization (ESI) - mass spectrometry (MS) with a QTOF-2 mass spectrometer (Micromass, UK). The samples were injected through a slightly pressurized borosilicate capillary (Thermo Scientific, USA). Capillary and cone voltages were set to 900 and 35 V, respectively. The mass range of 50–2000 Da was calibrated with a mixture of sodium iodide and cesium iodide (Sigma-Aldrich, USA). MS-MS was performed by selecting a 2–3 Th mass window in the first quadrupole and the precursors fragmented at collision energies between 25 and 35 eV to achieve enough structural information. Data acquisition and processing were performed with the MassLynx version 3.5 package (Micromass, UK).

### Vaccine doses and schedule

All monkeys were screened for Abs against IL-15 proteins, and considered naive with respect to the antigen when specific Abs were undetectable by enzyme-linked immunosorbent assay (ELISA, titer < 1:50; see methods below). The animals were immunized subcutaneously at several sites of the interscapular region with 200 μg of mhIL-15 in a total volume of 0.5 mL adjuvanted with either Alum (1.8 mg/mL, Brenntag Biosector, Denmark), Montanide ISA-51 VG (50:50 v/v, SEPPIC, France) or IFA (50:50 v/v, Sigma, USA). Three immunizations were performed, spaced 1 month between the first and second, and 2 months between the second and third. In the case of the Alum-adjuvanted mhIL-15 group, there were two additional immunizations at months 8 (fourth dose) and 18 (fifth dose) after the third inoculation.

Blood samples were collected before beginning the scheme (pre-immune), 15 days after each immunization, and 3 and 6 months after the third dose. In the case of animals immunized with Alum-adjuvanted mhIL-15, samples were also taken 10 months after the fourth dose. Complement was inactivated by incubating the sera at 56 °C for 30 min and the sample were then stored at -20 °C until used. Group serum pools were used to evaluate the recognition of native or simian IL-15 by ELISA. For this purpose, equal volumes of serum from animals of the same group were mixed.

### ELISA for serum anti-IL-15 Abs

Specific Abs titers against IL-15 and the recognition of native or simian IL-15 by immune sera were measured through an ELISA as indicated below. EIA 96-well plates (Costar, USA) were coated overnight at 25 °C with 1 μg/mL of mhIL-15, simian IL-15 (previously obtained at the laboratory of CIGB, Havana, Cuba) or native IL-15 (R&D, USA) in PBS pH 7.4. After 3 washes with 0.05 % Tween 20 (Calbiochem, Germany) in PBS, the plates were blocked with 1 % bovine serum albumin (BSA, Sigma-Aldrich, USA) in PBS for 1 h at 37 °C, followed by 3 washes. PBS, 0.05 % Tween 20 and 0.01 % BSA-diluted sera or pool of sera (starting dilution 1:1000 or fixed dilution 1:4000, respectively) were added to wells and incubated for 2 h at 37 °C. Wells were then washed 3 times and incubated with anti-monkey IgG (Fc specific)-peroxidase Ab (Sigma, USA) diluted 1: 10 000 in PBS. After incubating for 1 h at 37 °C, the plates were washed 5 times and incubated with 100 μL of substrate solution (Ultra 3, 3′, 5, 5′-Tetramethylbenzidine Liquid Substrate System, Thermo Scientific, USA) for 15 min. The reaction was stopped by adding 50 μL of 2 N sulphuric acid solution (R&D Systems, USA) and the absorbance at 450 nm was measured with an ELISA plate reader (Biotrak GE, Healthcare, USA). The 450 nm absorbance value corresponding to a PBS sample was subtracted from all the obtained diluted serum readings. Ab titer was considered as the highest serum dilution yielding at least twice the value of the optical density (OD) at 450 nm of the pre-immune serum from each animal. The data were processed using the GraphPad Prism program v6.05 (GraphPad Software, Inc.).

### Effect of serum on the proliferation of CTLL-2 cells

To evaluate the neutralizing capacity of individual or pooled samples, twofold serial dilutions of heat-inactivated sera (starting dilution 1:100 or 1:25 respectively) were performed in 96-well plates (Costar, USA) in a volume of 30 μL of RPMI medium supplemented with 10 % FBS. MAB247 and MAB202 (R&D, USA), which are commercially available neutralizing anti-human IL-15 and anti-human IL-2 Abs respectively, were used as positive controls in a range of 1 μg/mL to 7.8 ng/mL (twofold serial dilutions). Then, previously washed CTLL-2 cells were added in amounts of 5 × 10^3^ cells/well in 50 μL. Afterwards, 300 pg/mL of native human IL-15 (R&D, USA) or recombinant simian IL-15 or 50 ng/mL of human IL-2 (R&D, USA) in a volume of 20 μL was added to each well, and the plate was incubated for 72 h at 5 % CO_2_ and 37 °C [35]. After 72 h, yellow tetrazolium MTT (3-(4, 5-dimethylthiazolyl-2)-2, 5-diphenyltetrazolium bromide, Sigma, USA) was added and the plates were further incubated for 4 h [36]. Finally, 100 μL of a solution containing 10 % sodium dodecyl sulfate (Merck, Germany), 0.1 N HCl (Sigma-Aldrich, USA) and 50 % isopropyl alcohol (Pharmco-AAPER, USA) were added per well. Plates were read at 578 nm on a Multiscan (Sensident Scan, Merck, Germany). Curve Expert Program V.1.3.80 (www.curveexpert.net/) was used to calculate the neutralizing titers of sera from immunized monkeys with the anti-IL-15 vaccine. These titers were expressed as the dilution of sera that is required for inhibiting the proliferation by at least 50 % (ID_50_). The data were graphed using the GraphPad Prism program v6.05 (GraphPad Software, Inc.).

### Inhibition of IL-15-mediated TNF-α production in synovial fluid cells from patients with RA

After obtaining written informed consent, synovial fluid from RA patients was extracted and incubated with 10 μg/ml hyaluronidase (Sigma, USA) for 45 min at 37 °C. Synovial fluid cells were obtained after centrifugation at 1200 g for 10 min. The cells were incubated in 96-well plates at 2 × 10^5^ cells per well either with serum (dilution 1:1000), or 60 ng/ml of native IL-15 (R&D, USA), or a combination of both. After 48 h of incubation, the supernatants were collected and stored at -70 °C until further evaluation. TNF-α concentration was determined by ELISA (R&D Systems, USA) according to the manufacturer’s instructions. The data were graphed using the GraphPad Prism program v6.05 (GraphPad Software, Inc.).

### Determination of macaque CD8^+^ and CD56^+^ cell populations from whole blood samples

One hundred microliters of blood containing 1 % Ethylenediaminetetraacetic acid (EDTA-Na_2_, Sigma-Aldrich, USA) as anticoagulant were gently homogenized with 2 mL of lysis solution (0.15 M NH_4_Cl (Sigma-Aldrich, USA), 1 mM KHCO_3_ (Sigma-Aldrich, USA) and 0.1 mM EDTA in 0.2 L of distilled water), keeping the samples in the dark for 15 min at 25 °C and mixing them every 5 min. After this time, the reaction was stopped by incubation on ice for 2 min, the cells were centrifuged at 1200 g for 5 min and the supernatant was discarded. The cell pellet was washed 3 times with 1 mL of cold PBS by gentle homogenization and centrifugation at 1200 g for 5 min. Afterwards, the cells were incubated on ice with 25 μL of Fluorescein isothiocyanate (FITC)-labelled anti-human CD8 monoclonal Ab clone 17D8 (Exalpha Biologicals, USA) diluted 1:3 and an anti-human CD56 monoclonal Ab clone MEM-188 (BioVendor, Czech Republic) diluted 1:25 in PBS, keeping the samples in the dark for 30 min. The cells were then washed 3 times as indicated above and the samples labeled with the anti-human CD56 Ab were incubated on ice for 30 min with anti-mouse IgG (whole molecule)-FITC Ab (Sigma, USA) diluted 1:30 in PBS. After washing the cells 3 times as described above, they were analyzed in a PARTEC Pass II flow cytometer (Partec, Germany) by collecting 20 000 events. The percentages of cells with CD8 and CD56 surface markers were obtained from the analysis of samples using FloMax software v2.4 (Partec, Germany).

### Statistical analysis

The Shapiro-Wilk and Leveneʼs tests were used to verify normality and homogeneity of variance. The comparison of the levels of TNF-α and the percentages of CD8^+^ and CD56^+^ cells was performed with Studentʼs *T* test for paired samples. Statistical significance was set at *p* ≤ 0.05. In all cases, the SPSS/PASW (Statistical Package for the Social Sciences/ Predictive Analytics Software) Statistics for Windows version 18 (Chicago: SPSS Inc.) was used.

## Results

### Characterization of mhIL-15 by MS

**Fig. 1:**
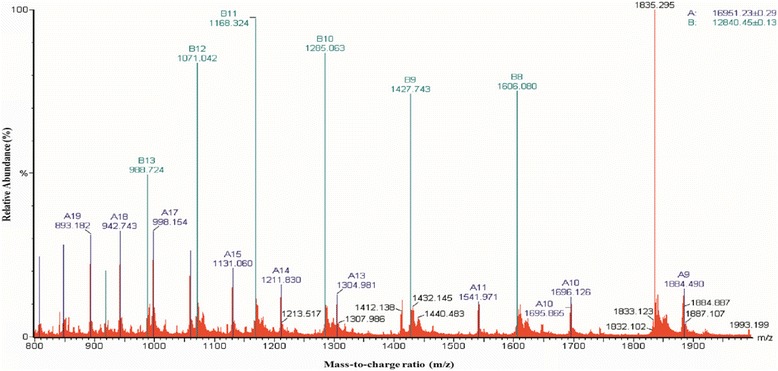
Mass spectrum of the intact IL-15 purified from *E. coli*. Signals corresponding to multi-charged ions are showed for the molecule (B_8_, B_9_, B_10_, B_11_, B_12_ and B_13_) and the myoglobin (A_9_, A_10_, A_11_, A_13_, A_14_, A_15_, A_17_, A_18_ and A_19_) used as an internal standard (Theoretical molecular mass 16951.50 Da)

The purification process described for the recombinant human IL-15 expressed in *E. coli* allowed a 95 % of protein purity [34]. This protein contains two disulfide bridges in its structure. Figure 1 depicts the spectrum of multiple charge ions of intact IL-15 obtained by ESI-MS, where a single species of protein outstands with a molecular mass of 12 840.45 ± 0.13 Da. This molecular mass is similar to the expected molecular mass of 12 840.59 Da obtained from the cloned DNA sequence corresponding to the presence of two disulfide bonds and an additional alanine residue in the N-terminal of protein.

Amino acid sequence of IL-15 was verified by ESI-MS peptide-analysis of trypsin and Glu-C tandem enzyme digestions. Table 1 shows the experimental molecular mass and the sequence assignments.

**Table 1:** Peptides generated by IL-15 digestion with trypsin/Glu-C enzymes observed by ESI-MS

Experimental m/z	Theoretical m/z	Charge	Error	Localization and amino acid sequence
565.73	565.75	1	0.02	^55^SGDASIHDTVE^65^
629.81	629.84	2	0.03	^1^ANWVNVISDLK^11^
747.32	747.34	2	0.02	^43^CFLLE^47^ ^30^SDVHPSC^36^K^37^
801.43	801.47	2	0.04	^48^LQVISLE^54^
441.64	441.64	2	0.00	^84^SGC^86^KEC^89^EE^91^
936.95	936.99	2	0.04	^66^NLIILANNSLSSNGNVTE^83^
955.95	956.00	2	0.05	^100^FLQSFVHIVQMFINTS^115^
1060.49	1060.52	2	0.03	^12^KIEDLIQSMHIDATLYTE^29^

Cys^43^-Cys^36^ and Cys^86^-Cys^89^ are linked by disulfide bonds

*m/z* mass-to-charge ratio

**Fig. 2:**
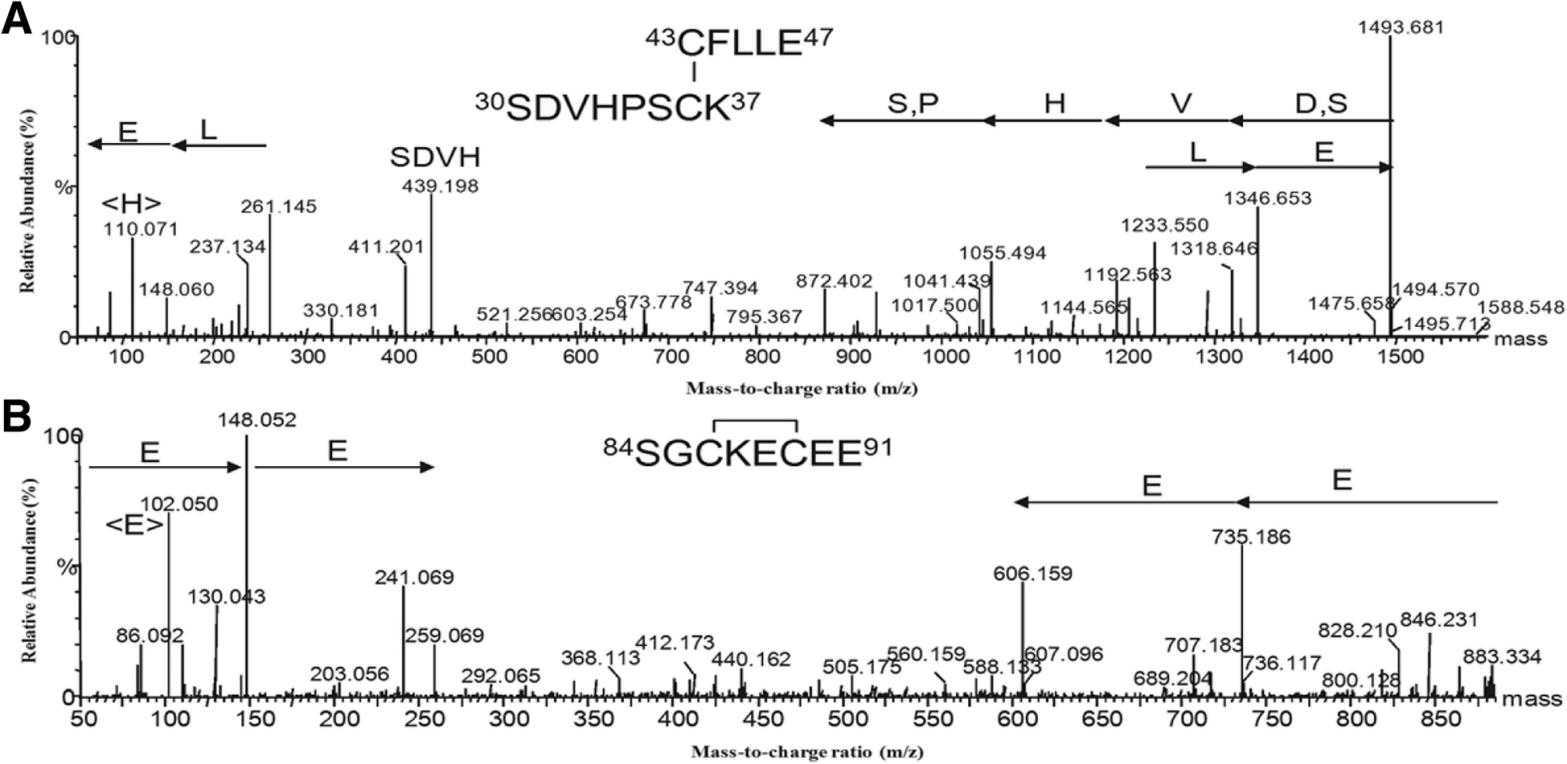
Fragment mass spectra of disulfide bonds containing peptides of mhIL-15 after tandem trypsin/Glu-C digestion. The disulfide bonds between cysteine 36–43: ^43^CFLLE^47^ ^30^SDVHPSC^36^K^37^ (**a**) and cysteine 86–89: ^84^SGC^86^KEC^89^EE^91^ (**b**) were shown

Sequencing of peptides m/z 441.64 and 747.34 (double charged) confirmed that the purified IL-15 contains disulfide bonds between Cys^36^- Cys^43^ and Cys^86^- Cys^89^ (Fig. 2a and b). This disulfide arrangement is different to the one described by Pettit for the native protein Cys^35^-Cys^85^ and Cys^42^ -Cys^88^ [37]. These results confirmed that the major fraction of the human IL-15 previously described by our group [34] was structurally modified with respect to the native protein.

### Abs response in NHP immunized with mhIL-15 using three different adjuvants

**Fig. 3:**
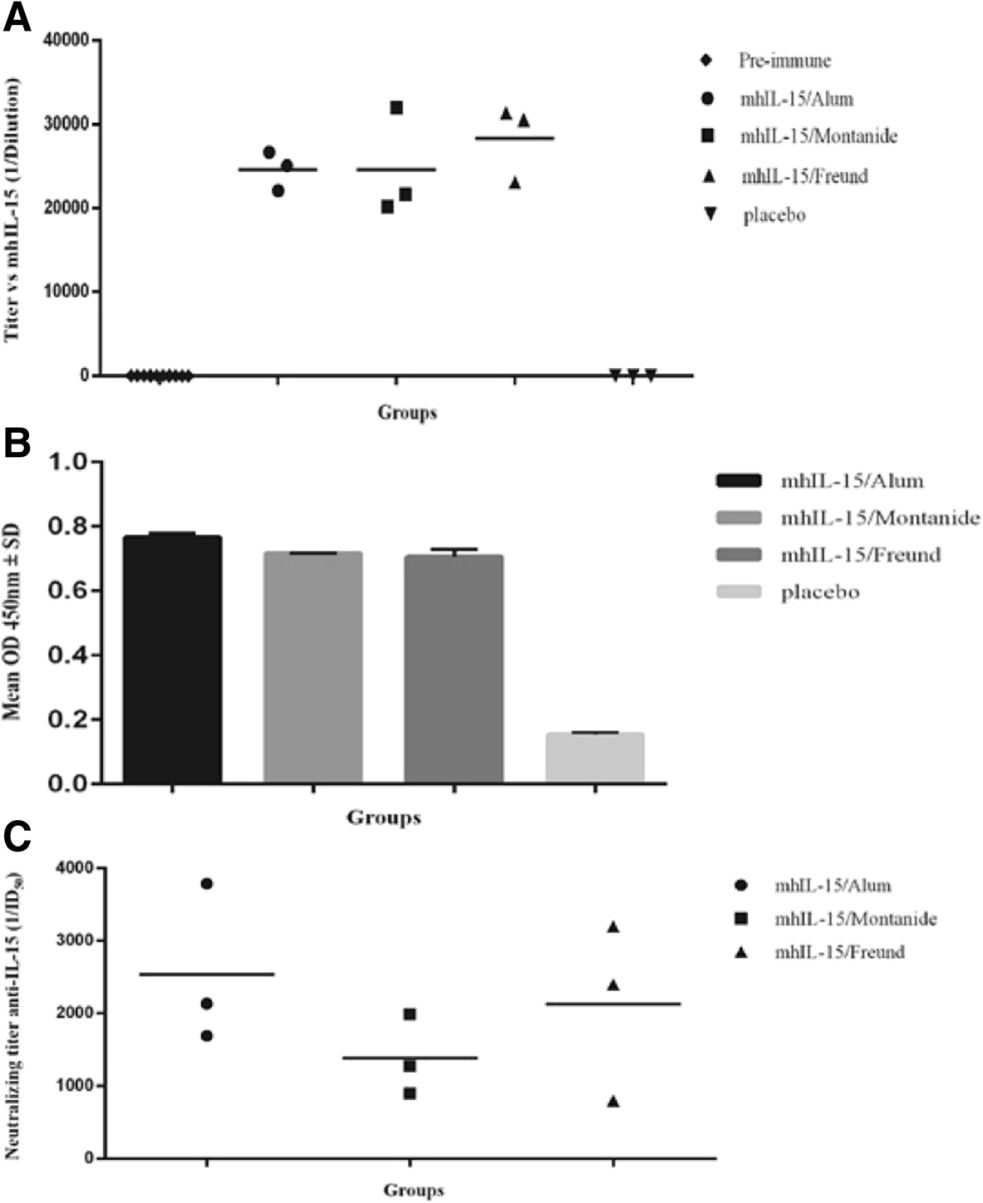
Abs response in macaques immunized with mhIL-15 corresponding to 15 days after the third immunization. **a** ELISA for Abs titers against IL-15. The plate was coated with 1 μg/ml mhIL-15 and the serum from each animal was evaluated in twofold serial dilutions (starting dilution 1:1000). All animals developed an Abs response, except pre-immune and placebo macaques. The line represents the mean values of Abs titers calculated from duplicate samples of individual monkeys (*n* = 3) corresponding to each experimental group. **b** ELISA for recognition of native IL-15 by Abs from sera of immunized macaques. The plate was coated with 1 μg/ml native human IL-15 and the pool of sera from each group was evaluated in fixed dilution, 1:4000. **c** Serum neutralization titers of macaques calculated from the data obtained in the CTLL-2 cell proliferation assay. The line represents the mean values of neutralization titers (expressed as 1/ID_50_) calculated from duplicate samples of individual animals (*n* = 3). The ID_50_ was determined by inhibiting of human IL-15-induced proliferation of CTLL-2 cells

Figure 3a depicts the anti-IL-15 Abs titers detected by ELISA in serum from macaques immunized with mhIL-15 in Alum, Montanide or IFA after the third immunization. Average titer was higher than 1:20000 in all groups, except in the pre-immune and placebo monkeys. The highest response was obtained in the group immunized with IFA eliciting an average titer of 1:28351; while in the Alum and Montanide groups the calculated average titer was 1:24660 and 1:24616, respectively.

Recognition of native IL-15 with the correct disulfide bridges was assessed by ELISA, but due to the few amount of available cytokine, we evaluated the pool of sera from each group. As shown in Fig. 3b, OD 450 nm values in immunized groups were, at least, four times higher than the one obtained for the placebo group. This result demonstrates that anti-IL-15 Abs generated by immunization with mhIL-15 recognized native IL-15 immobilized on the plate.

Also, neutralizing activity of sera from macaques immunized with mhIL-15 was evaluated by a CTLL-2 cell proliferation assay in presence of native human IL-15 [35]. We observed a neutralizing effect of sera corresponding to the third immunization in CTLL-2 cells. Figure 3c shows neutralizing titers for animals immunized with mhIL-15 using three different adjuvants. The higher neutralizing capacity was obtained in the group using Alum as adjuvant with titers of 1:1690, 1: 3790 and 1:2135, whereas sera from the Montanide group showed values of 1:897, 1:1987 and 1:1275. Sera from the group immunized with IFA showed higher neutralizing effect than the Montanide group with values of 1:800; 1:3200 and 1:2400. This result indicated that anti-IL-15 Abs produced by immunization with mhIL-15 inhibits native IL-15 biological activity in a cell line that proliferates in response to IL-15. Based on these results and some elements mentioned in the discussion section we chose Aluminum hydroxide as the adjuvant for vaccination with mhIL-15.

### Abs response in macaques immunized with mhIL-15 in Alum throughout the whole scheme

**Fig. 4:**
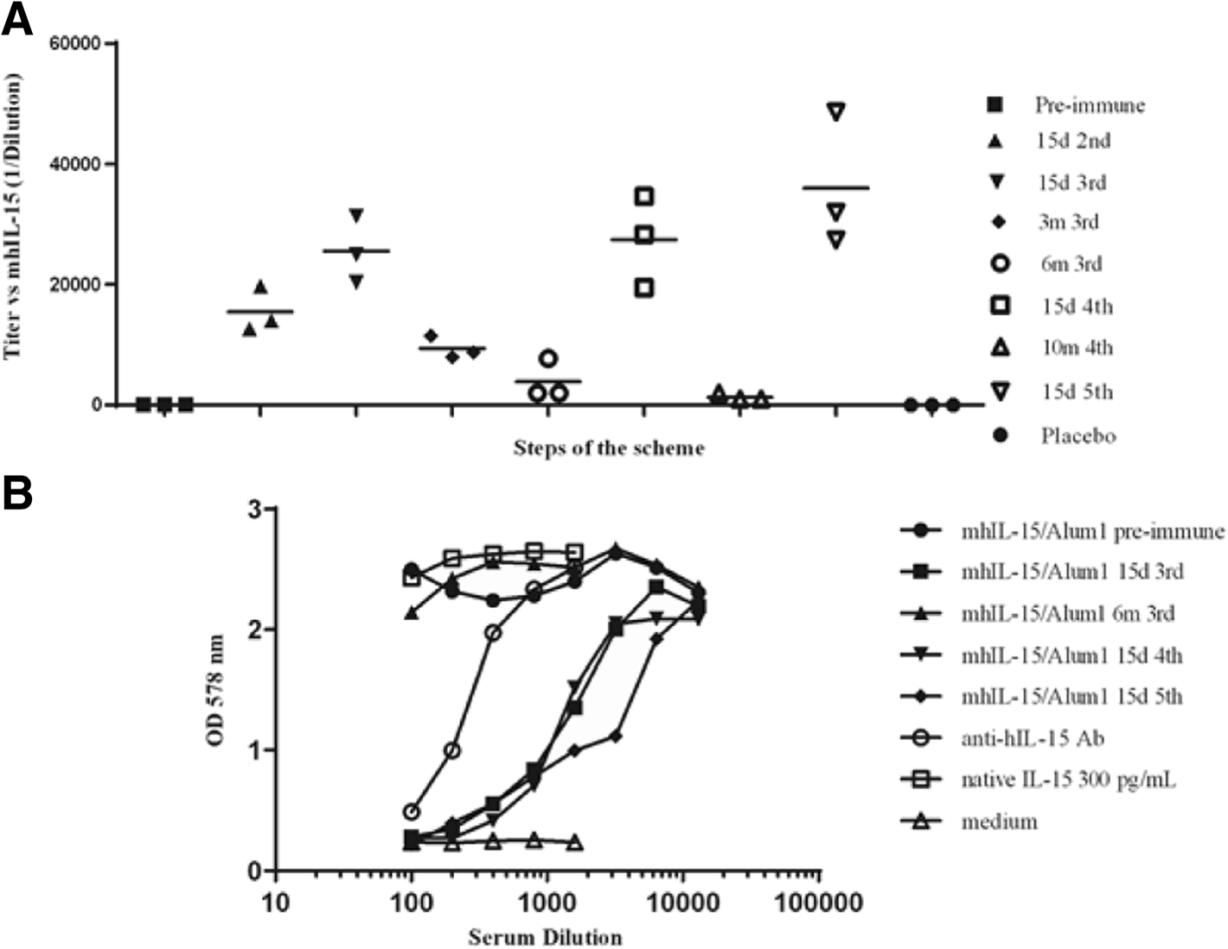
Abs response in macaques from the Alum group throughout the immunization scheme. **a** Titers of anti-IL-15 Abs detected by ELISA in macaques immunized with mhIL-15 adjuvanted in Alum. The plate was coated with 1 μg/ml mhIL-15 and serum was evaluated in twofold serial dilutions (starting dilution 1:1000). The Abs titers of pre-immune animals, placebo group and the monkeys immunized with mhIL-15 in Alum after the second, third, fourth and fifth immunization are shown. The line represents the mean values of Abs titers calculated from duplicate samples of individual animals (*n* = 3) during the scheme. **b** Proliferation assay in CTLL-2 cells with the serum of one animal from the Alum group. Cells were cultured in the presence of native IL-15, IL-15 plus dilutions of the pre-immune serum, or serum from each immunization (starting dilution 1:100), IL-15 plus serial dilutions of an anti-human IL-15 Ab and cells culture in medium without cytokine. Cell proliferation was evaluated by MTT staining. Similar results were obtained when the sera of other animals from the same group were assessed. d: days after immunization; m: months after immunization

Spaced immunizations were performed using Alum as adjuvant to address the duration of the Abs response. Figure 4a summarizes the course of the Abs response to the mhIL-15/Alum group throughout the scheme. Three months after the third immunization, the anti-human IL-15 Abs titers declined in more than 50 % with an average titer of 1:9475 while after the six months the average titer was 1:3930. In order to assess if we could reach levels of Abs titers similar to those obtained previously through re-immunization, we performed two additional immunizations. As shown in the graph, the Abs titers after the fourth immunization were very similar to those achieved after the third inoculation. Decreases in specific Abs titers were detected in the samples taken 300 days after the fourth dose (average titer 1:1333). However, in response to the fifth immunization we observed a full recovery of the Abs response, to levels similar to those obtained in previous immunizations (Fig. 4a).

Effects of sera from immunized macaques were evaluated using the CTLL-2 cell proliferation assay aiming to assess whether the titers of anti-IL-15 Abs obtained by ELISA corresponded with a neutralizing Abs response. Figure 4b depicts effects on the proliferation of the CTLL-2 cells of serum obtained from a macaque immunized with mhIL-15 in Alum. In correspondence with the results obtained by ELISA, highest neutralizing effects were present in sera of monkeys corresponding to 15 days after third, fourth and fifth immunizations. Neutralizing effects were smaller due to decreased titers of anti-IL-15 Abs as observed in a serum sample at 6 months after the third immunization. In agreement with the absence of Abs titers, pre-immune serum did not affect cell proliferation; however, commercial anti-human IL-15 neutralizing Abs inhibited proliferation in a dose-dependent manner. Similar results were obtained when the sera of other animals from the same group were assessed.

### Effect of the immune sera on the recognition and the activity of simian IL-15

**Fig. 5:**
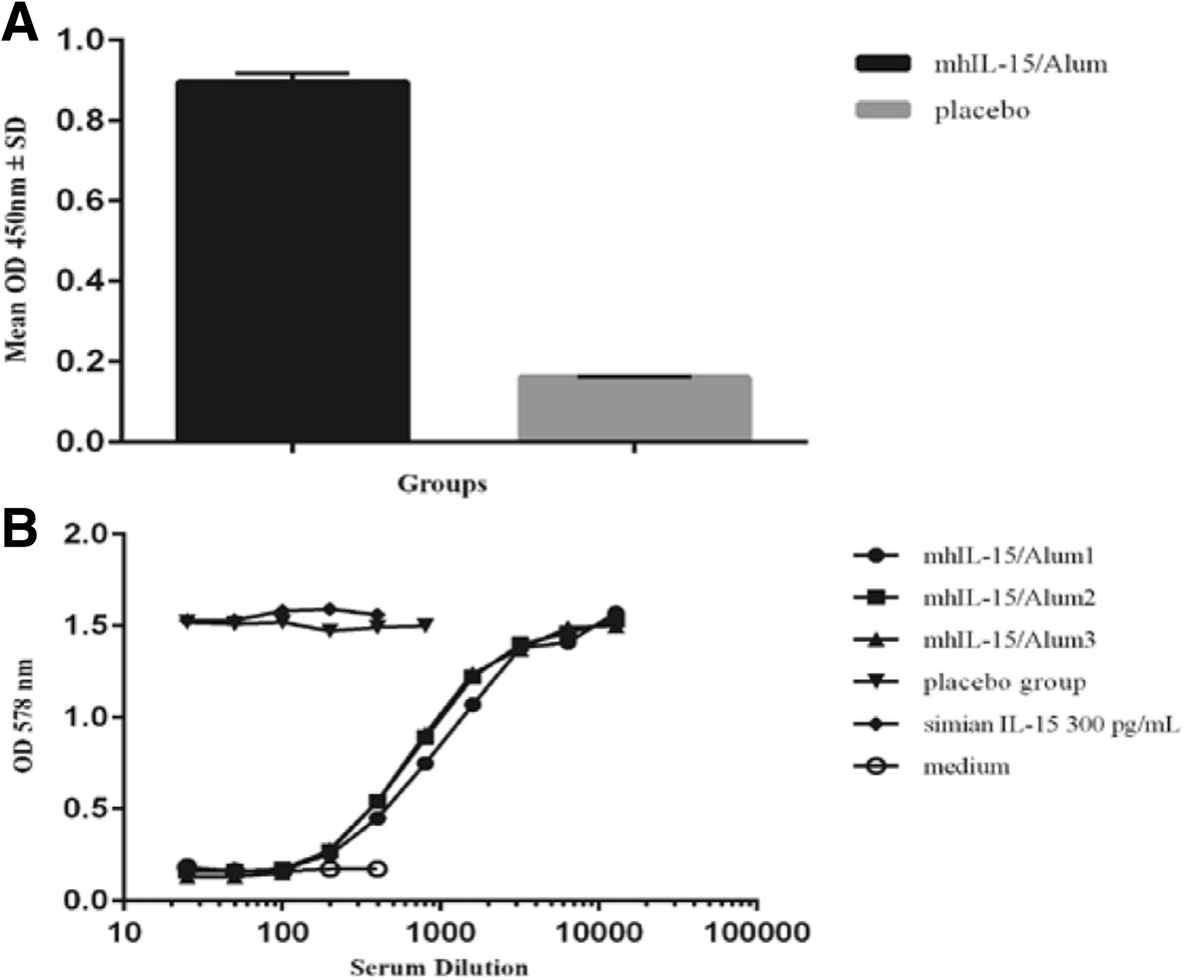
Recognition and neutralizing capacity of sera from the Alum group in presence of simian IL-15. **a** ELISA for recognition of simian IL-15 by Abs from sera of immunized macaques. The plate was coated with 1 μg/ml recombinant simian IL-15 and it was incubated with the pool of sera per group diluted 1:4000. **b** Inhibition of simian IL-15-induced proliferation of CTLL-2 cells by sera from the Alum group. Cells were cultured in the presence of simian IL-15, simian IL-15 plus serial dilutions of serum (starting dilution 1:25) or medium. Cell proliferation was evaluated by MTT staining. All tested sera correspond to 15 days after the third immunization

In order to evaluate the recognition of sera from immunized animals on self-IL-15 and their effect on the activity of simian IL-15, we obtained this cytokine by cloning its cDNA upon RNA isolated from PBMC of *Macaca fascicularis* in *E. coli* (unpublished results). First, recognition of simian IL-15 by Abs from sera of the Alum group was assessed by ELISA. As illustrated in Fig. 5a, the sera recognized simian IL-15 immobilized on the plate. The OD 450 nm values corresponding to the immunized groups were, at least, 5 times higher than that obtained from the placebo group. In addition, these sera inhibited the activity of simian IL-15 and showed a neutralizing effect in a dose-dependent manner (Fig. 5b). The calculated ID_50_ values were similar among the three animals (1:831, 1:635 and 1:636).

### Effect of the immune sera on IL-2 proliferative activity in CTLL-2 cells

**Fig. 6:**
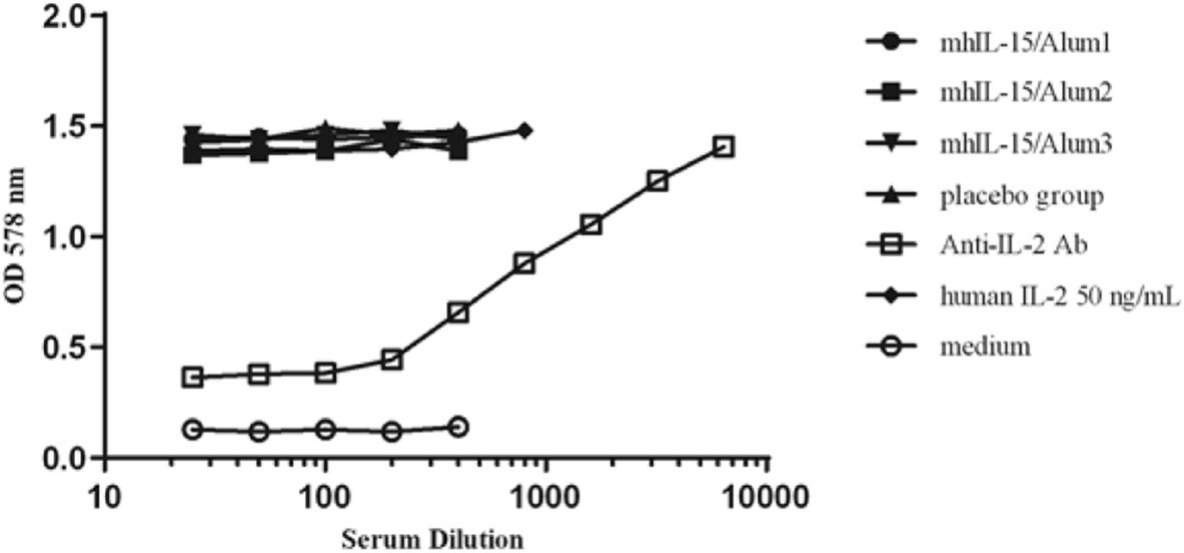
Effect of sera from Alum group on IL-2-dependent proliferative activity in CTLL-2 cells. Cells were cultured in presence of IL-2, IL-2 plus serial dilutions of sera (starting dilution 1:25), IL-2 plus serial dilutions of an anti-human IL-2 Ab or medium. Cell proliferation was evaluated by MTT staining. All tested sera correspond to 15 days after the third immunization

To study the specificity of the neutralizing activity of sera obtained from the Alum group, we assessed their effects on human IL-2-induced proliferation of CTLL-2 cells. As observed in Fig. 6, the Abs generated after mhIL-15-based immunization had no effect on human IL-2-induced proliferation of CTLL-2 cells; while the commercial neutralizing anti-human IL-2 Ab exhibited a dose-dependent inhibition.

### Effect of serum on TNF-α secretion in synovial cells from RA patients

**Fig. 7:**
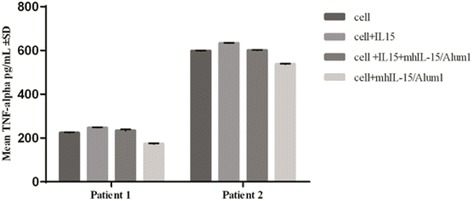
Effect of serum on TNF-α production mediated by IL-15 in synovial cells from RA patients. Synovial fluid cells were incubated with serum (fixed dilution 1:1000), or with 60 ng/ml of IL-15, or a combination of both. After incubation, supernatants were collected and levels of human TNF-α were quantified by ELISA. The tested serum corresponds to one animal from the Alum group and was taken 15 days after the third immunization

In order to assess activity of sera from immunized monkeys on other IL-15-induced biological functions, we measured effects on TNF-α secretion of one serum from Alum group corresponding to 15 days after the third immunization. For this purpose, we determined levels of IL-15 in synovial fluids from patients with RA [38] and we selected 2 patients with high concentration of this cytokine (≥25 pg/mL). In this experiment, we found that serum from an animal immunized with mhIL-15 in Alum inhibited TNF-α secretion induced by exogenous IL-15 in synovial cells. Additionally, this serum diminished significantly (p 0.05) the baseline levels of TNF-α secreted by these cells (Fig. 7).

### Effect of the vaccine on IL-15-dependent cell populations

Percentages of CD56^+^ and CD8^+^ cells before and after the fifth immunization were determined in order to study the effect of vaccination on IL-15-dependent cell populations, such as CD56^+^ NK and CD8^+^ T cells. These samples showed low and high titers of anti-IL-15 Abs, respectively.

Table 2 shows the quantitation of CD56^+^ and CD8^+^ cells determined by flow cytometry in samples from animals immunized with mhIL-15 in Alum. In this case, the samples did not exhibited statistically significant differences in percentages of IL-15-dependent cells with CD56 (p 0.658) and CD8 (p 0.684) markers.

**Table 2:** Percentages of CD56^+^ and CD8^+^ cells from whole blood of monkeys

	CD56^+^ cells (%)		CD8^+^ cells (%)	
Animals	Before the fifth immunization	After the fifth immunization	Before the fifth immunization	After the fifth immunization
mhIL-15/Alum_1_	13.8	13.9	20.2	16.0
mhIL-15/Alum_2_	5.9	6.7	31.3	37.2
mhIL-15/Alum_3_	5.9	3.5	17.9	20.4

### Clinical, behavioral, and laboratory parameters

During the observational time of 540 days, no differences were observed in immunized animals with respect to initial clinical observations, which included body weight, rectal temperature, and respiratory and cardiac rates. No lesions appeared at the inoculation site in immunized animals except for animals immunized with IFA. Additionally, no changes in any tested hematologic or blood biochemical parameters were observed.

## Discussion

In the current work, we evaluated a therapeutic vaccine composed of recombinant mhIL-15 as antigen. Our goal was to generate neutralizing antibodies which could inhibit IL-15 pathological effects. This vaccine could be used for treatment of autoimmune diseases, leukemia or transplant rejection, scenarios where uncontrolled expression of IL-15 is related to disease’s course [39–41]. At first, the structurally modified recombinant protein was expressed in the host *E. coli*. Recombinant mhIL-15 obtained by our group contains disulfide bridges between contiguous cysteines C^36^-C^43^ and C^86^-C^89^, unlike previous reported cysteines C^35^-C^85^ and C^42^-C^88^ for native IL-15 [37]. However, it is noteworthy that IL-15 obtained by us has an Alanine residue at the N-terminus of the protein before the initial codon. Consequently, location of cysteines in the mhIL-15 sequence is displaced in one residue compared with native IL-15 [37]. This structural modification is privileged due to the reducing environment in the cytoplasm of the bacterium, which does not favor formation of disulfide bonds. Additionally, we speculate that this modification can favor exposure of subdominant or cryptic epitopes that promote an effective Ab response against the native protein.

Selection of a suitable animal model is important for assessing immunogenicity of a recombinant protein. The NHP were selected because they are the specie of the greatest homology with humans in the amino acid sequence of IL-15, reporting a 97 % similarity between proteins of both species [42]. The latter is suitable for testing the concept that through vaccination with mhIL-15, a rupture of the immune tolerance can be achieved. Also, there is a high similarity between simian and human IL-15 regarding biological activity and recognition of receptor subunits [43] which could be advantageous to our approach. Thus, to assess the immunogenicity of IL-15 vaccine in NHP, we used three distinct adjuvants: Alum, Montanide and IFA. Immunization with mhIL-15 generates a response of anti-IL-15 Abs with titers superior to 1: 20000 after the third immunization in all groups. These results indicate a rupture of B cells tolerance as consequence of immunization and generation of specific Abs against the cytokine. Higher Abs titers were obtained in the group immunized with IFA, in correspondence with a potent immunostimulatory effect described for this adjuvant [44], although it also caused toxicity in every animal of this group, presenting septic ulcers at the site of inoculation. We are aware that this toxicity hinders its use in humans; nevertheless, we incorporated it in our scheme as an experimental control based on previous results not shown in this work.

On the other hand, Alum is the most widely used adjuvants in human vaccines [45], and its use elicits strong humoral immune responses primarily mediated by secreted antigen-specific Abs [46]. This constitutes a favorable element, considering that the aim of active immunotherapy against cytokine is to obtain blocking Abs from the immune system of the treated patient. In addition, Alum is a poor inducer of cell-mediated immune responses and is unsuitable for vaccines that require a strong cellular immune response [47]. Considering this elements and the fact that its use generated the response of anti-IL-15 Abs with higher neutralizing capacity, we selected Alum as adjuvant for active immunization with mhIL-15. However, taking into account the number of animals used in this study, it would be necessary to use additional animals per group to confirm the superiority of alum over other adjuvants like Montanide.

Due to the proliferation within some inflamed tissues, it is mandatory to avoid accumulation of T cells in most of the diseases where cytokines are chronically secreted. Therefore, it is critical when designing vaccines against cytokines to disrupt B-cell but not T-cell tolerance to self-cytokines, thereby eliciting production of neutralizing Abs in high titers [1, 23]. In this work, we showed that immunization with mhIL-15 generated neutralizing Abs against native IL-15. However, it is still necessary to demonstrate that immunization with this cytokine does not elicit a specific cellular response.

Our results indicated that Abs response was self-regulated when Alum was used as adjuvant. In our scheme, Abs titers begin to decrease after three months post-immunization, which could be due to limited production of these Abs by the activated B cells in absence of specific T helper cells [48]. Noteworthy, before a new re-immunization, the Abs response recovered to a similar extent of that achieved in previous immunizations.

These results suggest the generation of B memory cells, which are activated in response to a new immunization, so that it does not generate a response with sustained production of Abs, but rather a controlled response by immunization. Application of this vaccine would allow manipulation of treatments in such a way that may induce a controlled and not sustained Abs response against IL-15 in diseases such as RA which are characterized by periods of crisis and remissions [49]. Particularly, the frequency of boosters could be optimized in this disease monitoring both Abs titers by ELISA and neutralizing activity in immune sera.

Another important result was recognition and neutralization of native IL-15 by Abs generated by immunization. This element is favorable if one considers that our aim with an anti-IL-15 vaccine is to develop a specific Abs response against native IL-15 capable of neutralizing this cytokine overexpression and its pathological effect. A native IL-15 containing disulfide bonds in correct positions was used in all test. Our results indicate that Abs present in immune sera from macaques neutralized native IL-15 activity; showing correspondence between Abs titers and neutralizing effects.

Although our strategy did not include the introduction of foreign immunodominant T-helper epitopes into native structure of the cytokine nor the fusion of the self-protein to a carrier, we demonstrated that immunization with mhIL-15 generated a specific neutralizing Abs response against native IL-15. We speculate that structural modifications of the mhIL-15 could favor an exposure of dominant epitopes that cooperates with B cells in generating a specific Abs response against IL-15. In previous studies, our group found that at least fusion of mhIL-15 with P64K protein as a carrier generates an antibody response that does not neutralize native IL-15 (unpublished results), but we do not rule out the possibility that fusion to another protein such as Keyhole limpet hemocyanin (KLH), Virus-like particles (VLP) or Ovalbumin (OVA) could develop a neutralizing Abs response against IL-15.

On the other hand, the specificity of neutralizing sera was determined in presence of IL-2, considering that CTLL-2 cells also proliferate with this cytokine. Although receptors for IL-2 and IL-15 share the β and γ_c_ subunits and some biological functions due to redundant effect have been described for these proteins [50], in this study we demonstrate that sera generated by immunization with mhIL-15 does not inhibit IL-2-induced proliferation. In this cell line, no toxic effect caused by sera on CTLL-2 cells was observed, supporting the statement that the decrease on cell proliferation obtained in previous experiments with native IL-15, was due to the neutralizing capacity of Abs generated by immunization.

It has been proposed that an IL-15 antagonist could be useful for treating some autoimmune diseases in which IL-15 acts as proinflammatory cytokine. Particularly, high levels of IL-15 have been found in synovial fluids from RA patients [51], and it is known that in response to IL-15, synovial T cells secrete TNF-α directly and induce TNF-α synthesis by macrophages through cognate interactions [18, 52]. In this work, we demonstrated that immunization with mhIL-15 in Alum generates a neutralizing serum that slightly diminished levels of TNF-α in synovial cells, with or without IL-15 stimulation. Although, the number of tested patients is very small, these results suggest that this vaccine could be useful for treating RA taking into account that TNF-α is an important and validated target in RA, nevertheless additional experiments will be necessary to confirm this hypothesis.

Despite a high homology between human and simian IL-15 (97 % amino acid sequence identity), our results demonstrated that sera from animals immunized with mhIL-15 inhibited simian IL-15 biological activity in a CTLL-2 cell proliferation assay. This finding suggests that Abs against epitopes present in simian IL-15 were generated through vaccination, validating the use of this species as a model in evaluating the proposed strategy. In this sense, as security elements, we assessed the effects of immunization on cell populations that are important targets of IL-15, such as CD56^+^ NK and CD8^+^ T cells [53, 54]. We found that vaccination with a mhIL-15 adjuvanted in Alum did not affect the percentage of cells with CD8 and CD56 markers.

Although in human two phenotypically and functionally distinct peripheral blood NK cell subsets have been described based on the expression of CD56 and CD16 [55], in NHP the ability to investigate the role of NK cells in the models of disease has been greatly limited by the lack of appropriate phenotypic markers for this cellular population. In this study we used CD56 as a marker to assess NK cell. However, in previous studies in primates, the expression of CD56 was defined as a marker of a minor subset of NK cells [56–59]. To confirm that vaccination do not affects the number of entire NK cells, it would be necessary further experiments before carry out a clinical trial with this approach. For this purpose we could use the human NK receptor monoclonal Abs for NKp80 and NKG2A, alone or in combination with anti-CD16 monoclonal Ab to identify the entire NK cell population in NHP. While anti-NKp80 recognized virtually all NK cells in macaques, anti-NKG2A proved to be significantly more specific than anti-NKp80 for simian NK cells. Nevertheless, the same specific recognition of NK cells could be achieved with anti-NKp80 if used in combination with anti-CD16 [60].

This aspect is important in assessing the safety of the strategy we propose. While the goal is to apply this vaccine for therapeutic purposes in patients with elevated levels of IL-15, it is important to study a possible effect of immunization on the physiological activity of this cytokine in individual’s immune defense. Despite the fact that high affinity Abs against cytokines will not impair the physiology of normal tissues [47] and that anti-cytokine vaccination have been demonstrated to be safe in experimental and clinical studies [61–63], it would be necessary to demonstrate that immunization with IL-15 does not interfere with functionality of these cell populations.

In our research was not possible to study the variation on IL-15 levels in serum from immunized macaques because in physiological conditions exists many regulatory elements that allow the modest expression of IL-15 [64]. In fact, in serum from healthy controls as well as in healthy NHP, the median IL-15 value was less than 2 pg/mL [65, 66], so it is difficult to detect changes at this level. Therefore, it is tricky to study the variation on IL-15 levels in serum from the NHP used in the current work, although we agree that this is informative data and will be considered in the clinical trials of this therapeutic vaccine.

In parallel, all animals were monitored during the scheme by measuring temperature, body weight, heart rate and behavioral state. The biochemical parameters in blood were into the physiological limits established for these species. Only severe local lesions are observed in inoculation sites of animals immunized with IFA, some adverse events associated with use of this adjuvant [67]. As future purposes of this work, it is necessary to demonstrate effectiveness of this vaccine in an animal model of the disease and in humans through clinical trials. Nevertheless, the use of NHP as a model can guide us on the clinical potential of this cytokine in humans due to the high homology between these species.

## Conclusion

This study shows that the immunization with mhIL-15 using three different adjuvants: Alum, Montanide and IFA is able to generate specific neutralizing Abs against self-IL-15 in NHP. Interestingly, the highest neutralizing response was obtained in macaques immunized with mhIL-15 adjuvanted in Alum, although, it is necessary to confirm the superiority of this adjuvant over others with additional animals. The anti-IL-15 Abs elicited by the immunization were capable to recognize and neutralize the activity of native and simian IL-15. Additionally, the immunization did not affect the percentage of CD56^+^ NK and CD8^+^ T cells, nor the clinical signs or blood biochemical parameters of the monkeys. Furthermore, in cells from synovial fluid of two patients with RA, the anti-IL-15 Ab slightly inhibited the expression levels of TNF-α. In summary, the results presented in this paper demonstrate for the first time the immunogenicity and some safety aspects of a vaccine based on active immunization with mhIL-15 in healthy NHP. This strategy could be useful in the treatment of patients with disorders in which overexpression of IL-15 has been related with the course of the disease.

## Abbreviations

Abs: Antibodies
Alum: Aluminum hydroxide
BSA: Bovine serum albumin
CENPALAB: National Center for Animal Breeding
CIGB: Center for Genetic Engineering and Biotechnology
EDTA: Ethylenediaminetetraacetic acid
ELISA: Enzyme-linked immunosorbent assay
ESI-MS: Electro spray ionization/Mass spectrometry
FBS: Fetal bovine serum
FITC: Fluorescein isothiocyanat
ID_50_: Half-inhibitory dilution
IFA: Incomplete Freund’s Adjuvant
IL: Interleukin
IL-15: Interleukin-15
KLH: Keyhole limpet hemocyanin
mhIL-15: Modified human IL-15
MTT: (3-(4, 5-dimethylthiazolyl-2)-2, 5-diphenyltetrazolium bromide
NHP: Non-human primates
NK: Natural killer
OD: Optical density
OVA: Ovalbumin
PBS: Phosphate buffered saline
RA: Rheumatoid Arthritis
RP-HPLC: Reverse phase-high performance liquid chromatography
SEC: Size-exclusion chromatography
TNF-α: Tumour necrosis factor alpha
VLP: Virus-like particles
